# Supplementary material for: Kinesin-5-independent mitotic spindle assembly requires the antiparallel microtubule crosslinker Ase1 in fission yeast
Source: Nat Commun. 2017 May 17;8:15286. doi: 10.1038/ncomms15286 (PMC5442317; doi:10.1038/ncomms15286)
Supplement: Supplementary Information — Supplementary Figures, Supplementary Tables. [file ncomms15286-s1.pdf]

Supplementary Information

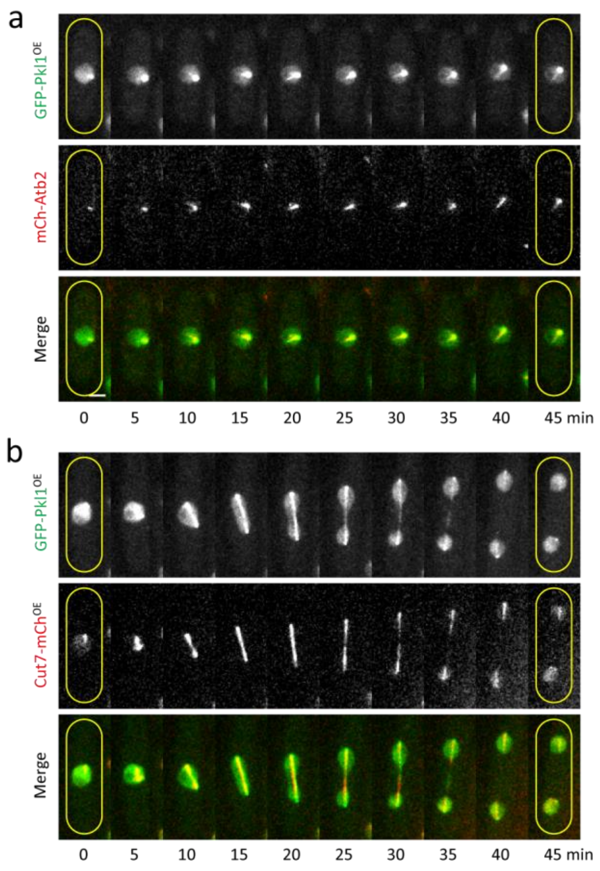

Supplementary Figure 1. Cut7 over-expression rescues monopolar spindles produced by Pkl1 over-expression.

a) Time-lapse images of a mitotic mCherry-Atb2 cell overexpressing GFP-Pkl1 from mitosis onset to spindle breakdown. Each frame corresponds to 5 minutes interval. Scale bar, 2 µm. **b)** Time-lapse images of a mitotic cell co-overexpressing GFP-Pkl1 and Cut7-mCherry. Each frame corresponds to 5 minutes interval.

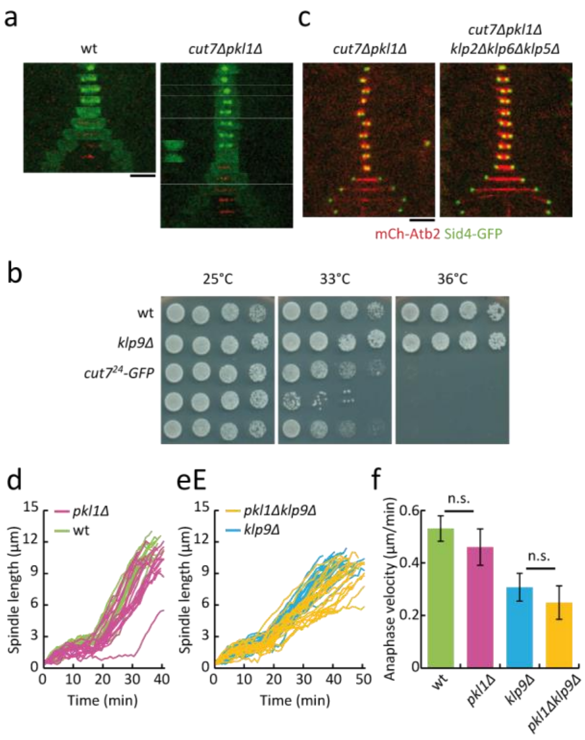

Supplementary Figure 2. No other kinesin can replace Cut7 for spindle assembly.

a) Time-lapse images of wild-type (wt) and *cut7*Δ*pkl1*Δ cells expressing Klp9-mCherry and Cdc13-GFP from mitosis onset to spindle breakdown. Each frame corresponds to 3 minutes interval. **b)** Serial dilution (4-fold) assay showing the genetic interaction of *klp9*Δ or *klp9* over-expression with the *cut7*^{24} strain. Plates were incubated 2-3 days at the specified temperatures. **c)** Time-lapse images of *cut7*Δ*pkl1*Δ and *cut7*Δ*pkl1*Δ*klp2*Δ*klp6*Δ*klp5*Δ cells expressing mCherry-Atb2 and Sid4-GFP from mitosis onset to spindle breakdown. Each frame corresponds to 3 minutes interval. **d)** Comparative plot of spindle length dynamics of wild-type (green curves; n=18) and *pkl1*Δ cells (pink curves; n=17). **e)** Comparative plot of spindle length dynamics of *klp9*Δ (blue curves; n=23) and *pkl1*Δ*klp9*Δ cells (yellow curves; n=20). **f)** Bar plot showing anaphase spindle elongation velocity of wild-type (green; n=18), *pkl1*Δ (pink; n=17), *klp9*Δ (blue; n=23) and *pkl1*Δ*klp9*Δ cells (yellow; n=20). ns, not statistically significant; *, p<0.001. Data show mean ± s.d. and Student t-test p-value. Scale bars, 2 μm.

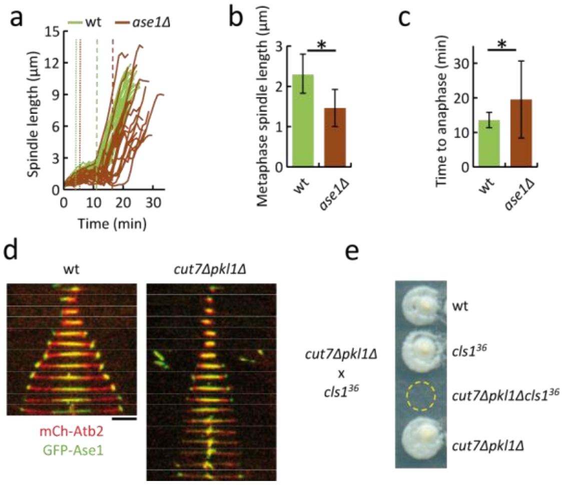

Supplementary Figure 3. Ase1 plays a role in spindle assembly.

a) Comparative plot of spindle length dynamics of wild-type (green curves; n=20) and *ase1*Δ cells (brown curves; n=20). Dotted vertical line corresponds to phase I to phase II transition; dashed vertical line corresponds to phase II to phase III transition. **b)** Bar plot showing metaphase spindle length at anaphase transition, reported by Cdc13-GFP degradation, of wild-type (green; n=56) and *cut7*Δ*pkl1*Δ cells (red; n=25). *, p<0.001. **c)** Bar plot showing the time from MT nucleation to Cdc13-GFP degradation of wild-type (green; n=56) and *cut7*Δ*pkl1*Δ cells (red; n=25). *, p<0.01. **d)** Time-lapse images of wild-type (wt) and *cut7*Δ*pkl1*Δ cells expressing mCherry-Atb2 and GFP-Ase1 from mitosis onset to spindle breakdown. Each frame corresponds to 3 minutes interval. **e)** Tetrad dissection of *cut7*Δ*pkl1*Δ cells crossed to *cls*^{136} cells. Data show mean ± s.d. and Student t-test p-value. Scale bars, 2 μm.

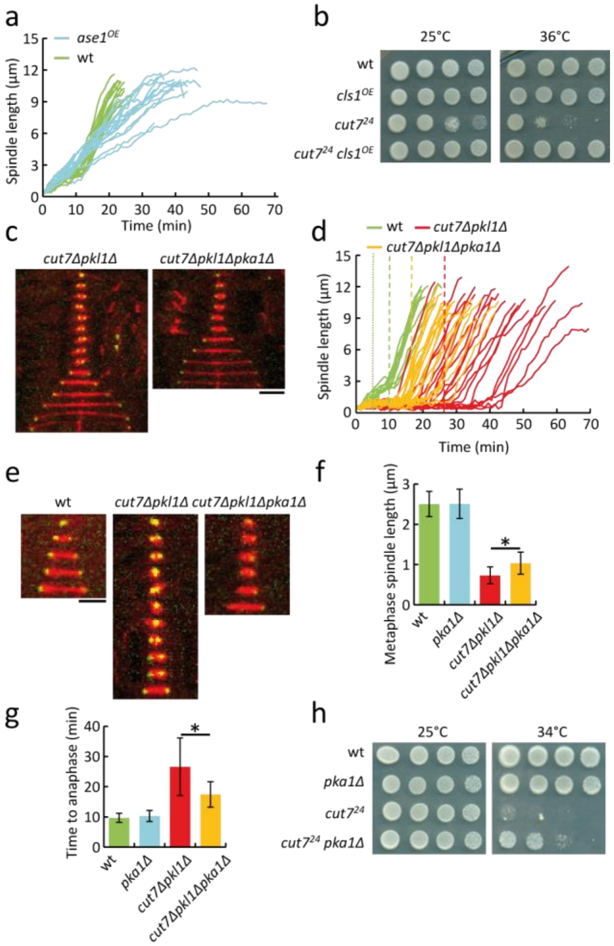

Supplementary Figure 4. MT stabilization by *pka1*Δ partially rescues the metaphase delay of *cut7*Δ*pkl1*Δ cells.

a) Comparative plot of spindle length dynamics of wild-type (green curves; n=15) and Ase1 overexpressing cells (light blue curves; n=15). **b)** Serial dilution (4-fold) assay showing the genetic interaction of Cls1 over-expression with the $cut7^{24}$ strain. Plates were incubated 2-3 days at the specified temperatures. **c)** Time-lapse images of *cut7*Δ*pkl1*Δ and *cut7*Δ*pkl1*Δ*pka1*Δ cells expressing GFP-Atb2 and Sid4-mCherry from mitosis onset to spindle breakdown. Each frame corresponds to 3 minutes interval. **d)** Comparative plot of spindle length dynamics of *cut7*Δ*pkl1*Δ (red curves; n=20) and *cut7*Δ*pkl1*Δ*pka1*Δ cells (orange curves; n=20). Dotted vertical line corresponds to phase I to phase II transition; dashed vertical line corresponds to phase II to phase III transition. **e)** Time-lapse images of wild-type, *cut7*Δ*pkl1*Δ and *cut7*Δ*pkl1*Δ*pka1*Δ cells expressing GFP-Atb2 and Sid4-mCherry from mitosis onset to beginning of anaphase. Each frame corresponds to 3 minutes interval. **f)** Bar plot showing spindle length at the anaphase transition, derived from the graphs shown in D, of wild-type (green; n=10), *pka1*Δ (light blue; n=10), *cut7*Δ*pkl1*Δ (red; n=20) and *cut7*Δ*pkl1*Δ*pka1*Δ cells (orange; n=20). *, $p<0.01$. **g)** Bar plot showing the time of anaphase transition, derived from the graphs shown in D, of wild-type (green; n=10), *pka1*Δ (light blue; n=10), *cut7*Δ*pkl1*Δ (red; n=20) and *cut7*Δ*pkl1*Δ*pka1*Δ cells (orange; n=20). *, $p<0.01$. **h)** Serial dilution (4-fold) assay showing the genetic interaction of *pka1*Δ with the $cut7^{24}$ strain. Plates were incubated 2-3 days at the specified temperatures. Data show mean ± s.d. and Student t-test p-value. Scale bars, 2 µm.

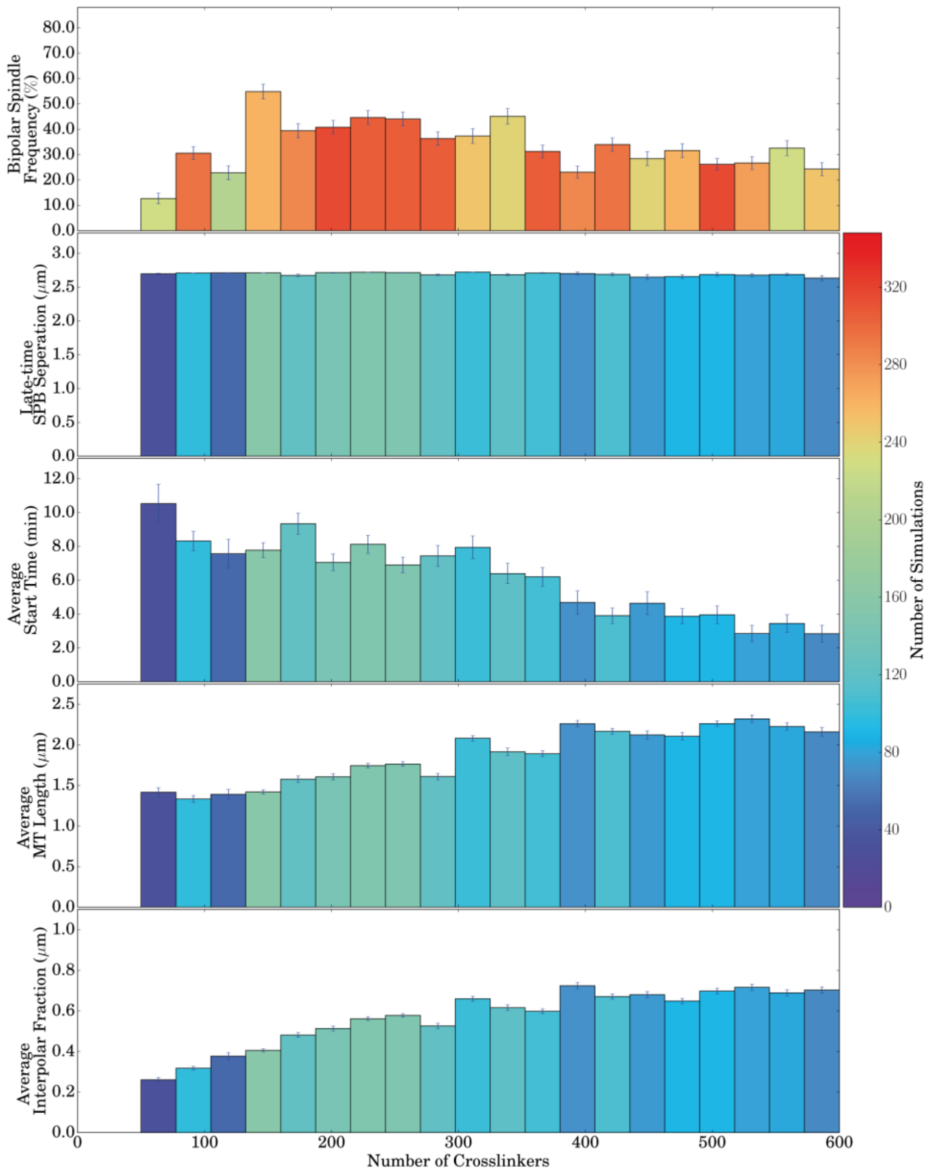

Supplementary Figure 5. Effects of varying number of Ase1 molecules.

Top, percentage of simulations in which a bipolar spindle forms. Subsequent plots show measurements only of simulations which form bipolar spindles. Average late-time SPB separation; average time at which spindle assembly initiates; average MT length; and average fraction of MTs in the interpolar bundle. Color bar on the right shows the number of simulations in each bar.

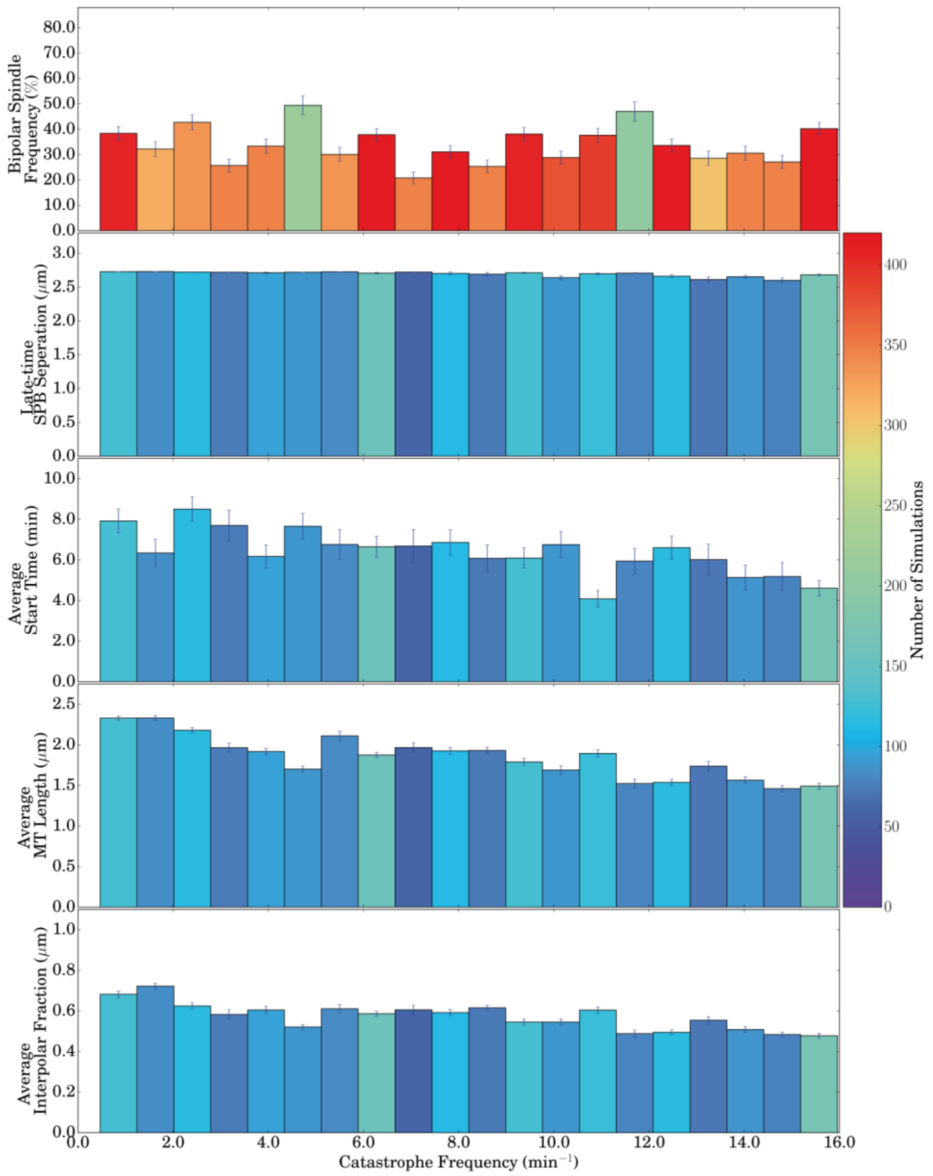

Supplementary Figure 6: Effects of varying MT catastrophe frequency.

Top, percentage of simulations in which a bipolar spindle forms. Subsequent plots show measurements only of simulations which form bipolar spindles. Average late-time SPB separation; average time at which spindle assembly initiates; average MT length; and average fraction of MTs in the interpolar bundle. Color bar on the right shows the number of simulations in each bar.

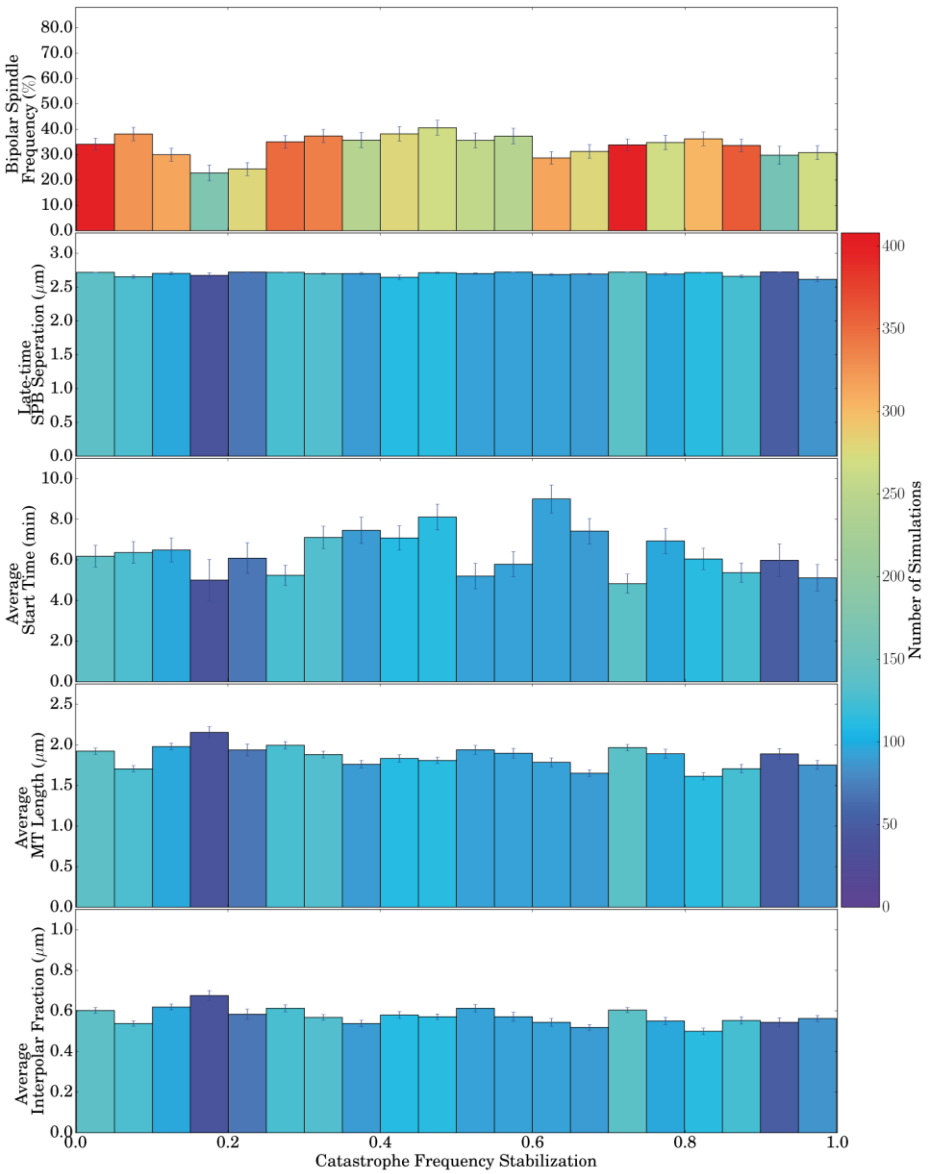

Supplementary Figure 7. Effects of varying MT catastrophe frequency stabilization.

Top, percentage of simulations in which a bipolar spindle forms. Subsequent plots show measurements only of simulations which form bipolar spindles. Average late-time SPB separation; average time at which spindle assembly initiates; average MT length; and average fraction of MTs in the interpolar bundle. Color bar on the right shows the number of simulations in each bar.

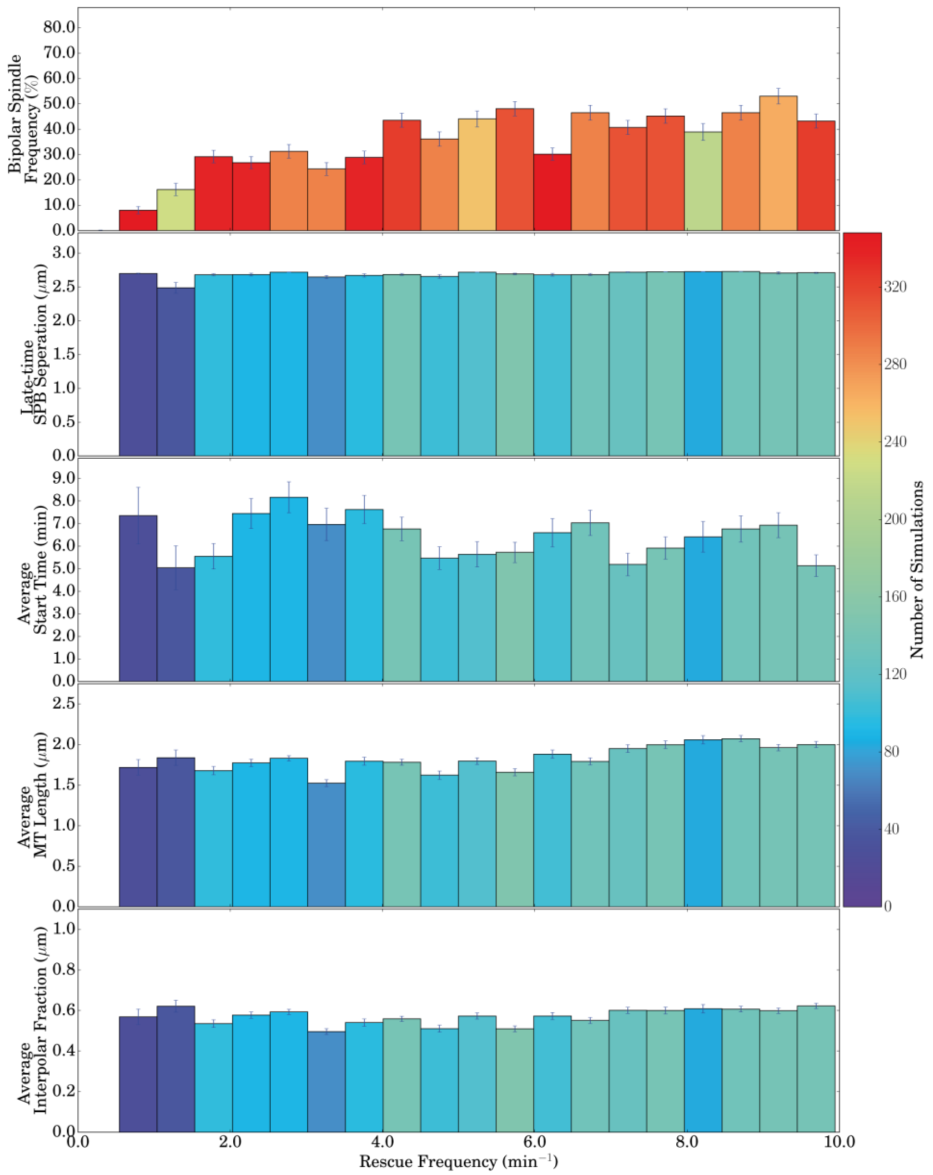

Supplementary Figure 8. Effects of varying MT rescue frequency.

Top, percentage of simulations in which a bipolar spindle forms. Subsequent plots show measurements only of simulations which form bipolar spindles. Average late-time SPB separation; average time at which spindle assembly initiates; average MT length; and average fraction of MTs in the interpolar bundle. Color bar on the right shows the number of simulations in each bar.

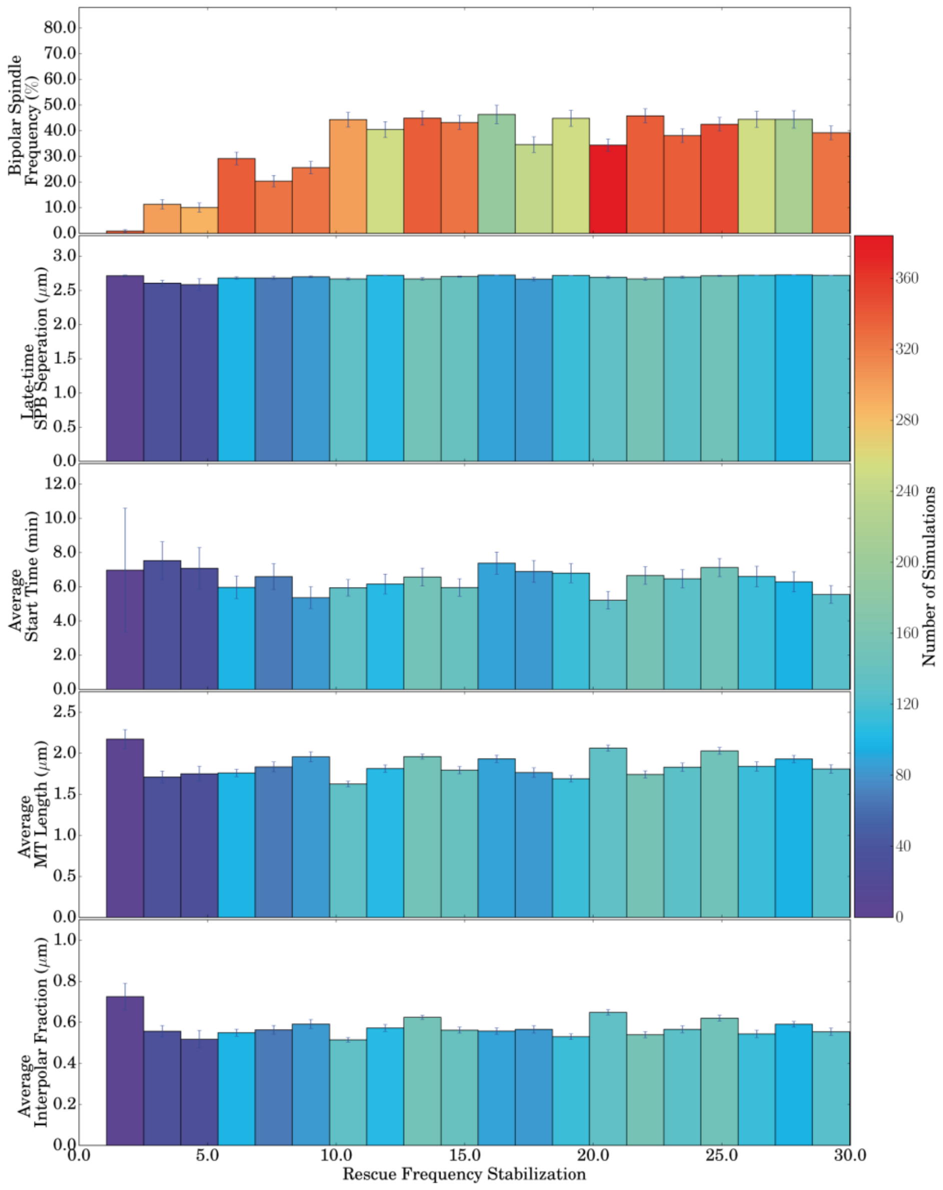

Supplementary Figure 9. Effects of varying MT rescue frequency stabilization.

Top, percentage of simulations in which a bipolar spindle forms. Subsequent plots show measurements only of simulations whichform bipolar spindles. Average late-time SPB separation; average time at which spindle assemblyinitiates; average MT length; and average fraction of MTs in the interpolar bundle. Color bar on the right shows the number of simulations in each bar.

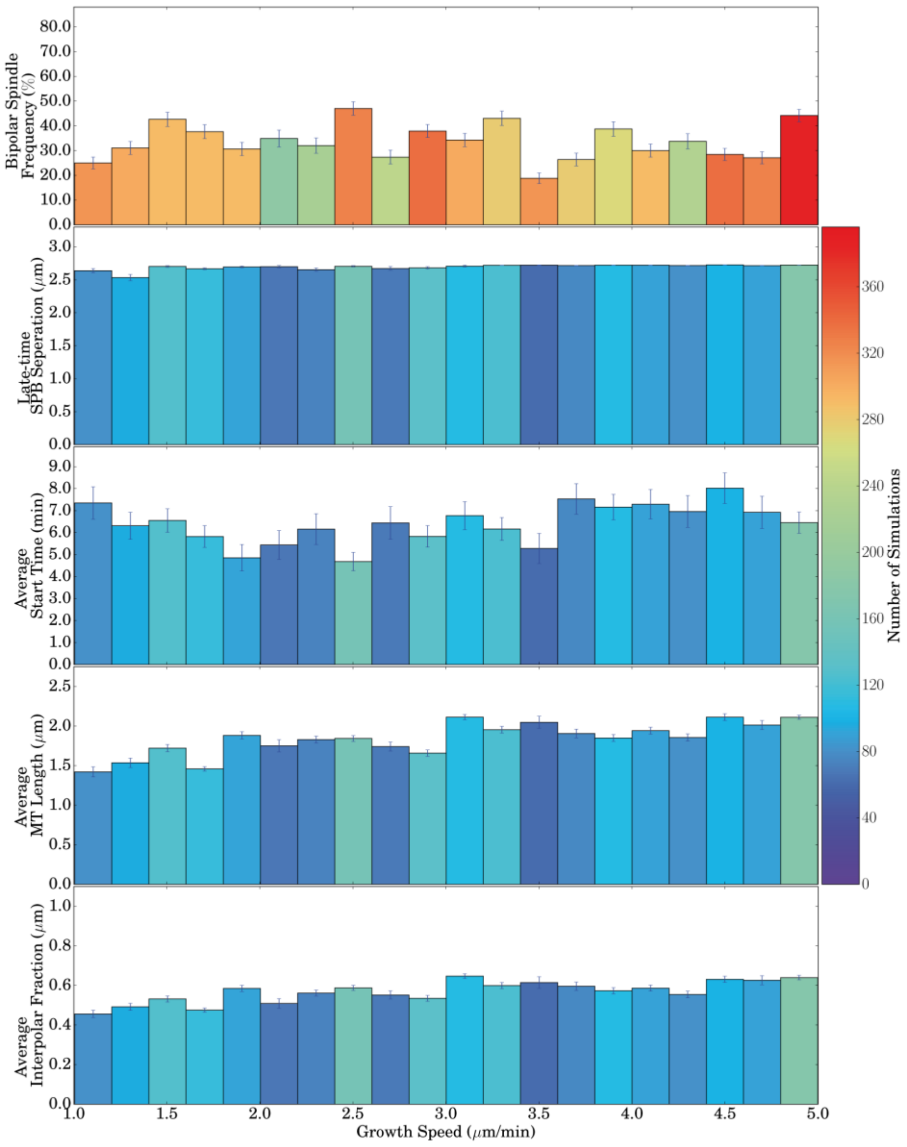

Supplementary Figure 10. Effects of varying MT growth speed.

Top, percentage of simulations in which a bipolar spindle forms. Subsequent plots show measurements only of simulations which form bipolar spindles. Average late-time SPB separation; average time at which spindle assembly initiates; average MT length; and average fraction of MTs in the interpolar bundle. Color bar on the right shows the number of simulations in each bar.

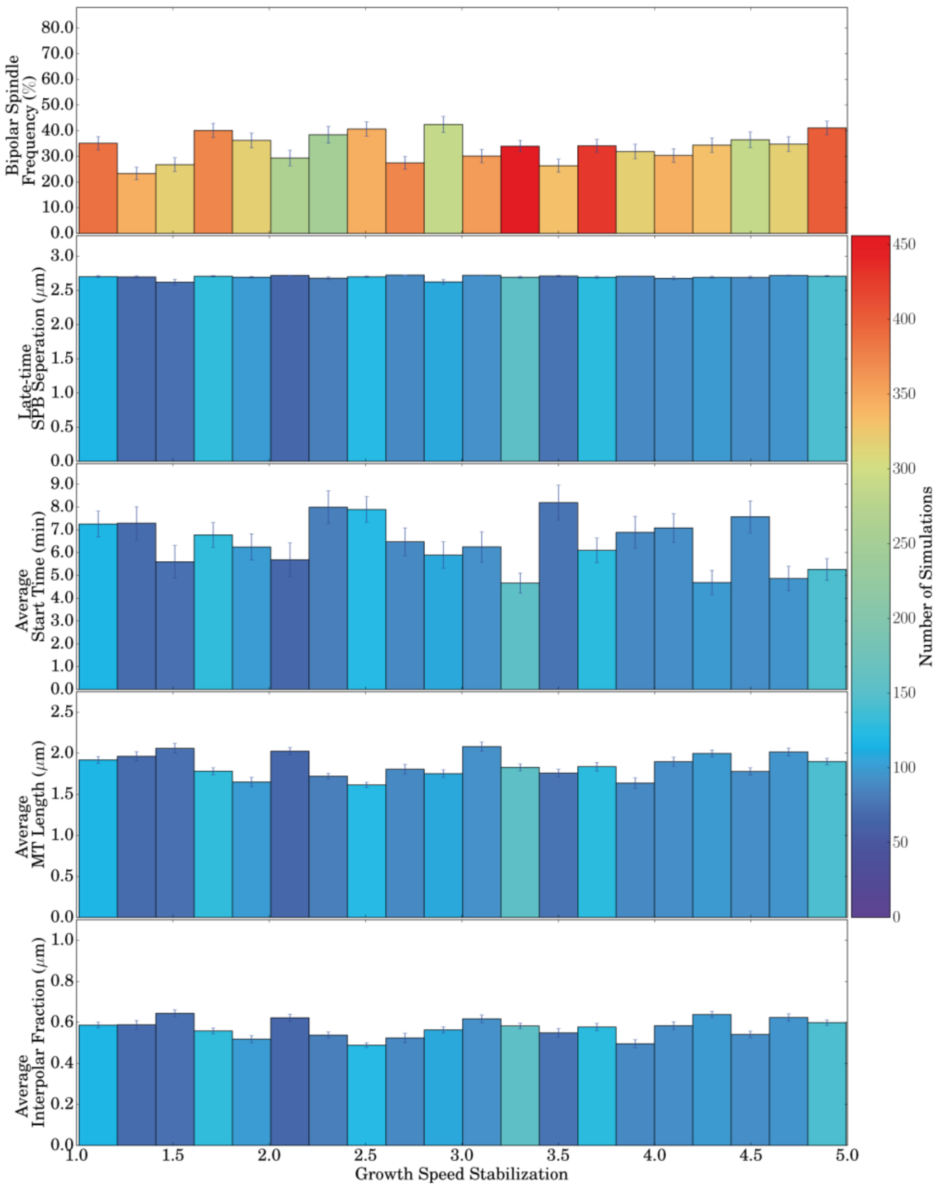

Supplementary Figure 11. Effects of varying MT growth speed stabilization.

Top, percentage of simulations in which a bipolar spindle forms. Subsequent plots show measurements only of simulations whichform bipolar spindles. Average late-time SPB separation; average time at which spindle assembly initiates; average MT length; and average fraction of MTs in the interpolar bundle. Color bar onthe right shows the number of simulations in each bar.

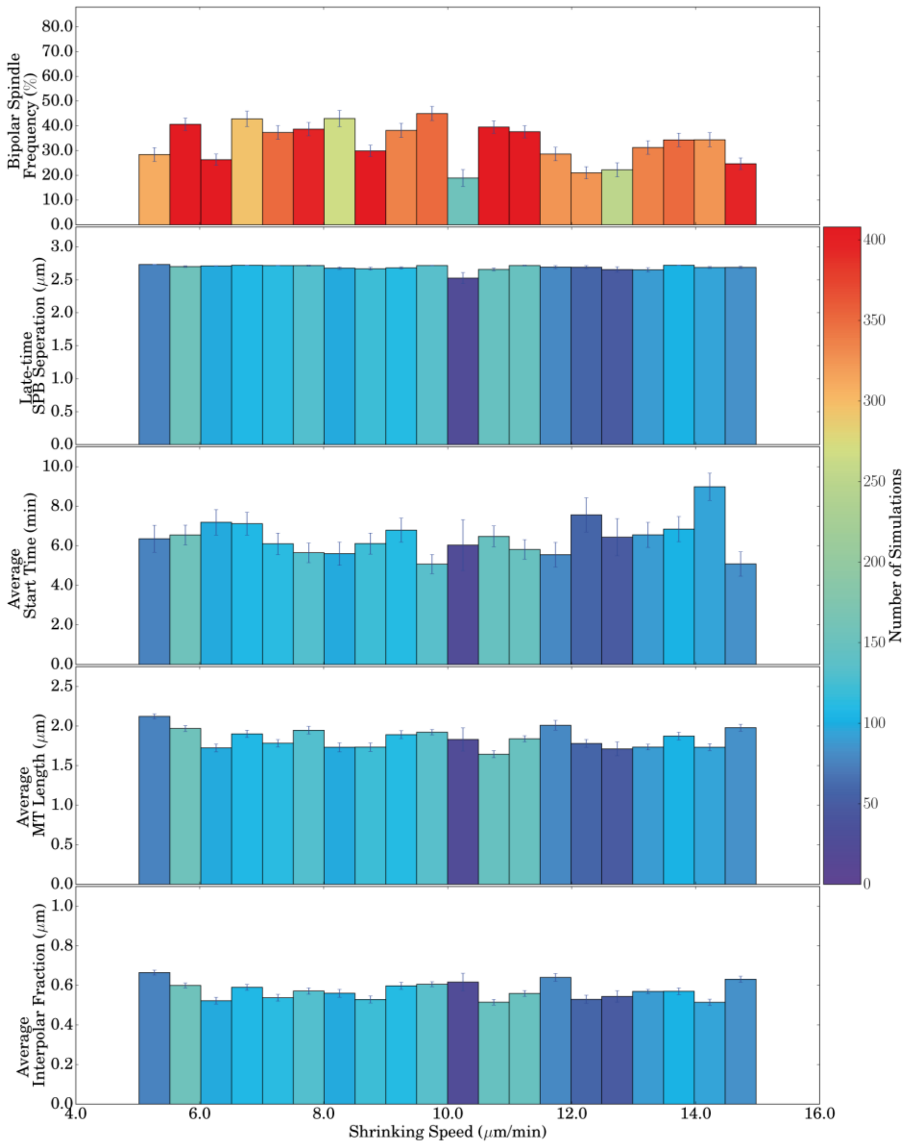

Supplementary Figure 12. Effects of varying MT shrinking speed.

Top, percentage of simulations in which a bipolar spindle forms. Subsequent plots show measurements only of simulations which form bipolar spindles. Average late-time SPB separation; average time at which spindle assembly initiates; average MT length; and average fraction of MTs in the interpolar bundle. Color bar on the right shows the number of simulations in each bar.

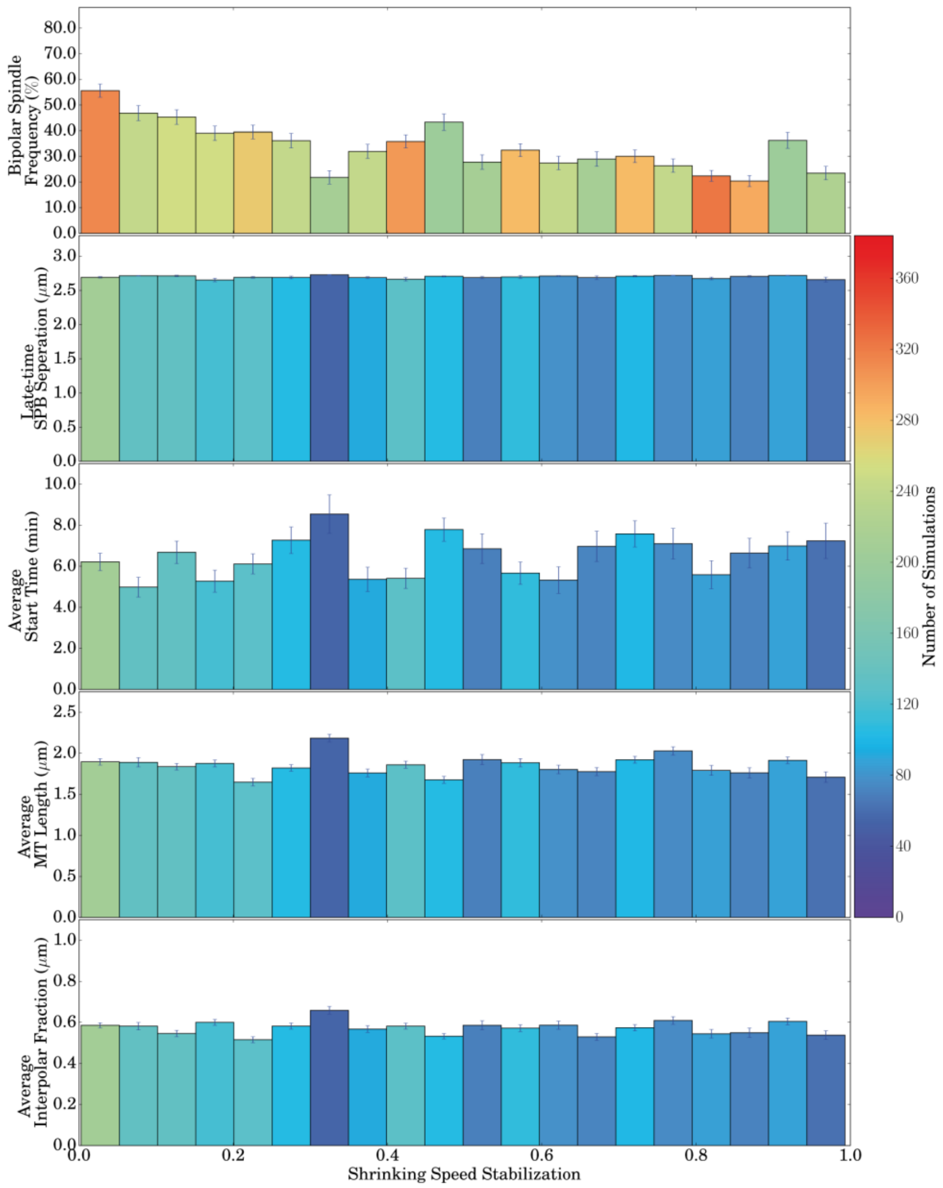

Supplementary Figure 13. Effects of varying MT shrinking speed stabilization.

Top, percentage of simulations in which a bipolar spindle forms. Subsequent plots show measurements only of simulations whichform bipolar spindles. Average late-time SPB separation; average time at which spindle assembly initiates; average MT length; and average fraction of MTs in the interpolar bundle. Color bar on the right shows the number of simulations in each bar.

Supplementary Table 1.
Geometric, SPB, and MT parameters.

Parameter	Symbol	Value	Notes
Nuclear envelope radius	R	1.375 μm	Kalinina et al. (2012)
MT diameter	σ_{MT}	25 nm	Alberts et al. (2008)
MT angular diffusion coefficient	D_θ	Varies with MT length	Kalinina et al. (2012)
Force-induced catastrophe constant	α_c	0.5 pN^{-1}	Dogterom and Yurke (1997)<br>Janson et al. (2003)
Asymptotic wall force	f_w	2.5 pN	Range 0 − 14.8, ∞ pN<br>Derenyi et al.(2002); Lim et al. (2007)
Membrane tube radius	f_{tube}	87.7 nm	Der´enyi et al. (2002); Lim et al. (2007)
**Spindle pole bodies**			
Diameter	σ_{SPB}	0.237 μm	Ding et al. (1993)
Bridge size	–	75 nm	Ding et al. (1993)
Tether rest length	R_0	50 nm	Flory et al. (2002); Muller et al. (2005)
Tether spring constant	K_0	0.67 pN/nm	Typical value of protein spring constant<br>cf. Blackwell et al. (2017a)
Number of MTs per SPB	N_{MT}	14	Ding et al. (1993)
Translational diffusion coefficient	D_t	4.5×10^{-4} $\mu m^2/s$	Blackwell et al. (2017a)
Rotational diffusion coefficient	$D_{\theta,spb}$	0.017/s	Blackwell et al. (2017a)
**Dynamic instability**			
MT growth speed	$v_{g,0}$	4 μm/min	Range 1–5 μm/min (Estimated)
MT shrinking speed	$v_{s,0}$	6.7 μm/min	Range 5–15 μm/min Estimated
Catastrophe frequency	$f_{c,0}$	6.07 /min	Range 0, .4–16 /min (Estimated)
Rescue frequency	$f_{r,0}$	0.71 /min	Range 0–10 /min (Estimated)
Growth speed stabilization	s_{vg}	1.5	Range 1–5, set to 1 when modeling Cls1 deletion
Shrinking speed stabilization	s_{vs}	0.1	Range 0–1, set to 1 when modeling Cls1 deletion
Catastrophe frequency stabilization	s_{fc}	0.1	Range 0–1, set to 1 when modeling Cls1 deletion
Rescue frequency stabilization	s_{fr}	20	Range 1–30, set to 1 when modeling Cls1 deletion
Stabilization length	s_l	25 nm	Estimated
Minimum MT length	L_{min}	50 nm	Value chosen for numerical stability
MT Stall force	f_s	14.6 pN	Dogterom and Yurke (1997)

Supplementary Table 2.
Ase1 paramenters.

Parameter	Symbol	Value	Notes
Available molecules	N_{tot}	250	Range 50-600; Carpy et al. (2014)
Association constant	K_a	90.9/μM/site	Cochran et al. (2004)
One-dimensional effective concentration	$c_{c,2}$	0.4/nm	Lansky et al. (2015)
Spring constant	K_c	0.2047 pN/nm	Lansky et al. (2015)
Diffusion constant (solution)	D_{free}	4.5 μm^2/s	Bancaud et al. (2009)
Singly bound diffusion constant	D_{sb}	0.1 μm^2/s	Lansky et al. (2015)
Doubly bound diffusion constant	D_{db}	0.0.0067 μm^2/s	Same as the singly bound hopping rate; Blackwell et al. (2017a)
Singly bound off-rate	k_1	0.1/s	Kapitein et al. (2008)
Doubly bound off-rate	k_2	0.05/s	Lansky et al. (2015)
Polar affinity	P_{aff}	0.33	Kapitein et al. (2008)
Unbinding load sensitivity	λ	0.01626	Blackwell et al. (2017a)
